# Teleophthalmology in Practice: Lessons Learned from a Pilot Project

**DOI:** 10.2174/1874431101711010020

**Published:** 2017-08-22

**Authors:** Haleh Ayatollahi, Aynaz Nourani, Taleb Khodaveisi, Hossein Aghaei, Mehrdad Mohammadpour

**Affiliations:** 1 Department of Health Information Management, School of Health Management and Information Sciences, Iran University of Medical Sciences, Tehran, Iran; 2 Eye Research Center, Rassoul Akram Hospital, Iran University of Medical Sciences, Tehran, Iran; 3 Farabi Eye Hospital, Eye Research Center, Tehran University of Medical Sciences, Tehran, Iran

**Keywords:** Ophthalmology, Optometry, Telemedicine, Remote consultation, Cataract, Teleophthalmology

## Abstract

**Introduction:**

Ophthalmology is a medical specialty which may benefit from using telemedicine and teleophthalmology services. Such services are significantly important in the poor, remote, and impassable geographical areas, where there is no access to the ophthalmology services and ophthalmologists. This study aimed to design and implement a teleophthalmology system using the method of store-and-forward.

**Methods:**

The study was conducted in 2015 and consisted of two main phases. The first phase was based requirement analysis, and in the second phase, after designing the prototype, an initial usability testing was undertaken in a teaching hospital. The participants of the study were 10 optometrists and 10 ophthalmologists (cornea specialists). For each phase of the research, a questionnaire was used to collect data, and the collected data were analyzed using descriptive statistics.

**Results:**

In this study, users’ requirements were initially investigated. Then, the teleophthalmology system was designed based on the literature review and the results derived from the requirements’ analysis. Finally, usability testing showed that the users were relatively satisfied with the system.

**Conclusion:**

According to the results, it can be concluded that the teleophthalmology technology can be used in the country by optometrists and ophthalmologists to improve eye health care services and to prevent the prevalence of curable eye diseases.

## 1 INTRODUCTION

In the recent years, information and communication technology (ICT) has been developed and applied to different areas of health care. Using this technology, health care services have changed to be more accessible and effective, especially when distance is a major concern. In fact, to overcome geographical health disparities, telemedicine has emerged as a discipline to improve healthcare services across the globe [1, 2]. Telemedicine services can be used in different areas of healthcare, such as diagnosis, treatment, prevention, education, and research [3], and have been applied in different medical specialties, such as oncology, dermatology, radiology, and pathology [4].

Ophthalmology is another medical specialty which may benefit from using telemedicine and teleophthalmology services. Such services are significantly important in the poor, remote, and impassable geographical areas, where there is no access to the ophthalmology services and ophthalmologists. In these areas, due to the prevalence of eye diseases and a lack of access to the medical facilities, patients may suffer from low vision or blindness. The use of this technology can help to facilitate examining, diagnosing, and managing eye diseases, sharing information between the optometrists and the specialists, conducting research, *e*-learning programs and professional development [5].

The use of telemedicine services in the specialty of ophthalmology can also help to increase the speed and quality of eye care services, and can reduce unnecessary and time consuming visits across the country [6-8]. As ophthalmology is an area, in which medical imaging plays an important role in making diagnostic decisions, eye images can be sent *via* telemedicine technology to facilitate making a diagnosis [9]. The rapid transmission of data and images is one of the most important aspects of teleophthalmology which helps doctors to be able to consult and make a decision very quickly. This, in turn, can improve the quality of patient care, particularly in the remote areas [10, 11].

The teleophthalmology services can also be used for public eye screening and diagnosing common eye diseases, such as cataract and refractive error. These services are delivered *via* the internet and a patient or a physician is able to contact a specialist through videoconferencing or web-based information systems [5]. It is notable that three methods; namely, store-and-forward, synchronous or videoconferencing, and hybrid are used in teleophthalmology [4, 12, 13]. Among them, videoconferencing requires internet bandwidth and the costs of its equipment are relatively high in addition to the low quality of images; therefore, it might not be suitable for diagnosing eye diseases which require high-quality color images [14]. Teleophthalmology mostly adopts the store-and forward-method, followed by interactive services, such as videoconferencing. The hybrid method includes both store-and-forward and real-time communication methods [15]. It is notable that a teleophthalmology system, like any other web applications should be designed to meet users’ specific needs and to achieve this, end-users must be involved in the design process [16]. Otherwise, adopting the existing systems used in other communities might not be a successful approach [17].

In Iran, uncorrected refractive errors and cataract are the main causes of visual impairment [18, 19] and some geographic areas receive limited eye care services mainly due to the economic status [20]. Therefore, to control the eye diseases in the country, it has been recommended to pay more attention to clinical screening, especially during primary eye care services, such as optometry. Moreover, the use of teleophthalmology and modern information technology tools and protocols has been suggested [21]. Although several studies have been conducted regarding the application of telemedicine technology in the country [22-25], it was the first time that a teleophthalmology system was developed using the method of store-and-forward. It is expected that the eye care services and saving costs can be gained directly or indirectly by using the system.

## 2 MATERIALS AND METHODS

This study was undertaken in 2015 and consisted of two main phases. The first phase was requirement analysis in which users’ requirements were investigated. In the second phase, the system was developed based on the results derived from the first phase, and the usability testing was conducted. Due to the prevalence of cataract in the country, it was selected as an eye disease to be able to pilot the prototype of the system. The setting of the research was a teaching hospital.

The system designed in this study aimed to connect two groups of users, namely optometrists and ophthalmologists. In the first phase of the study, 10 optometrists and 10 ophthalmologists (cornea specialists) participated. The method of convenience sampling was used to select the participants. The ophthalmology clinic was one of the busiest clinic and more than 100 patients were monthly visited by the ophthalmologists of this clinic. In the second phase of the study, 10 system users (5 optometrists and 5 cornea specialists) evaluated the usability of the system.

To determine users’ requirements, a questionnaire was designed based on the literature review [26-29]. The questionnaire included 53 closed questions which were divided into two main parts: a) required data elements for patients, optometrists and ophthalmologists and b) needed system features. There were two answers for each question, “necessary” (1) and “unnecessary” (0). At the end of the each part, there was an open-ended question which asked the participants to suggest further data elements or system features that might not be considered in the questionnaire. The content and the face validity of the questionnaire were checked by the experts. The reliability was confirmed by using a statistical test (KR-20 = 0.78).

The usability of the system and users’ satisfaction with the interface was evaluated using the standard Questionnaire for User Interaction Satisfaction (QUIS) version 5.5 provided by the University of Maryland [30]. It was a ten-point Likert scale questionnaire included five parts (27 questions); namely, overall reaction to the software (six questions), screen design and layout (four questions), terminology and systems information (six questions), learning (six questions), and system capabilities (five questions). The questionnaire was translated and its face validity was checked. According to the literature, the reliability of the questionnaire was (α = 0.94) [30]. This questionnaire was completed by 10 users (optometrists and ophthalmologists) who used the prototype of the system. To analyze the usability testing data, the Likert scale was divided into three levels “weak (1-3)”, “average (3.1-6)” and “good (6.1-9)”. Mean values were calculated for different parts of the questionnaire, and were reported for optometrists and cornea specialists separately. The final results were reported based on the three levels mentioned above.

## 3 RESULTS

As noted in the methods section, the questionnaires were distributed among 10 cornea specialists and 10 optometrists. The response rate among the cornea specialists and optometrists was (n=9, 90%) and (n=7, 70%), respectively. Regarding the age range, the highest frequency (n=7, 77.7%) among the cornea specialists and the optometrists (n=7, 100%) was related to the age group of 24-33. In terms of gender, more men than women were among the cornea specialists (n=6, 66.6%) and optometrists (n=5, 71.4%) and the highest frequency for working experience was related to 2-11 years for the cornea specialist (n=8, 88.8%) and for the optometrists (n=7, 100%).

After data analysis, the data elements identified “necessary” by at least 60% of the participants were selected to be included in the system. The data elements included four main categories; namely, patient identifying information, patient clinical information, optometrist's information, and specialist's information. In terms of the patient identifying information, all of the data elements except race (n=6, 37.5%), weight (n=7, 43.7%), height (n=4, 25%), and the patient’s address (n=8, 50%) were found necessary to be included in the system.

In terms of the patient clinical information, all items, such as disease history, family history, allergies, and eye images were considered essential (n=16, 100%). Regarding the optometrist's information, the medical council unique number of the optometrist (n=10, 62.5%) and his/her phone number (n=13, 81.2%) were found necessary. The last category was related to the specialist's information and all items, such as the medical council unique number (n=10, 62.5%), phone number (n=14, 87.5%) and the workplace address (n=10, 62.5%) were reported necessary. Similarly, all of the system features listed in the questionnaire, such as the possibility of uploading images (n=13, 81.2%), searching (n=16, 100%), prescribing (n=14, 87.5%), and reporting (n=14, 87.5%) were considered necessary by most of the specialists and optometrists.

The prototype of the system was designed based on the results derived from the first phase. To develop the prototype, ASP.net programming language was used. The system was designed to be used by both ophthalmologists and optometrists. The first page of the system was a log in page and included a general description about the system, rules and regulations, and some information about tele-ophthalmology. New users had to click and complete a registration form which included their name, surname, date of birth, sex, telephone number, email address, username and password. When completing the registration form, the type of the user either as an optometrist or as an ophthalmologist should be determined by the user. Finally, the registration process was completed and the account was activated by the site administrator.

The system had four main sections for optometrists that included logging in/out page, adding a new consultation request, checking the status of the previous requests, and viewing/revising personal information. An optometrist could add a new consultation request in the system. Each request required four steps to be completed. First of all, patient demographic (*e.g*., age, sex, name, *etc*.) and clinical data (*e.g*., disease history, family history, allergy, *etc*.) were entered into the system manually by the optometrist. Then, the eye images were attached in Fig. (**1**) and the optometrist’s opinion was added in the next step. The optometrist could save the eye images as part of the patient records without sending them to the specialist and the reasons for not sending the images to the specialist could also be entered into the system. The eye images could be obtained and uploaded in three different angles (center, temporal, and nasal). Finally, a specialist would be selected from a list of the ophthalmologists and a consultation request would be sent to him/her.

**Fig. (2):**
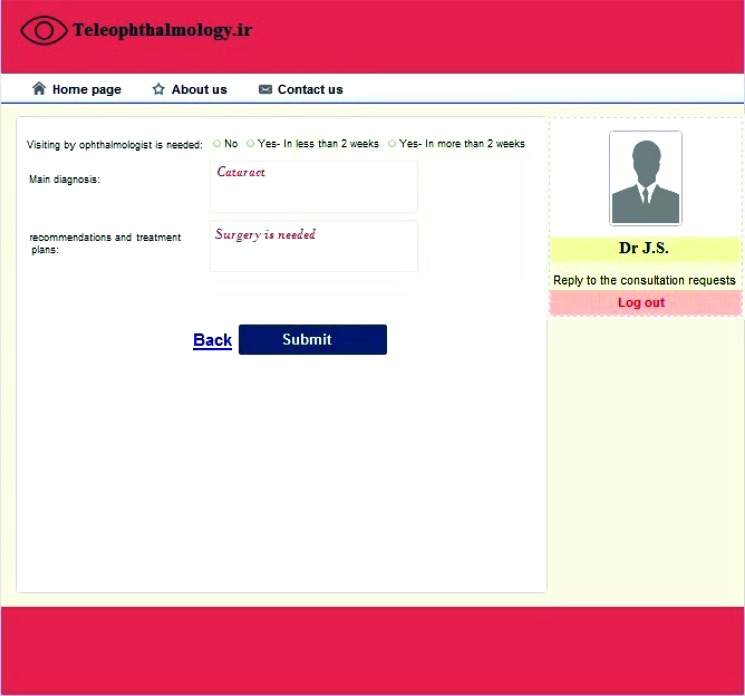
Reply to the consultation request.

Similar to the optometrists, the system had four sections for ophthalmologists that included logging in/out page, responding to the consultation requests, checking previous requests, and viewing/revising personal information. After sending a consultation request by an optometrist, the specialist could be notified by email and could see the consultation request on his/her personal panel. The consultation request was a read-only file, so the information could not be changed. The specialist was also able to review the patient demographic and clinical information, eye images, and the opinion of the optometrist, and could add his/her diagnosis and care plan by clicking on the “reply” button (Fig. **2**). The specialist could also recommend visiting the patient was necessary or not, and if it was necessary, it should be completed in less or more than two weeks. The diagnosis and the recommendations and treatment plans could also be entered by the specialist in the relevant boxes. Finally, the specialist's opinion would be sent back to the optometrist.

## 4 DISCUSSION

In many developing countries, teleophthalmology is being used to provide eye care to the underserved urban and remote rural population [15]. However, it was the first time in Iran that such a system was developed. In this study, since eye diseases are diverse, cataract was selected to be able to collect patients’ data and to evaluate the usability of the system. Cataract is an eye disease which has a high prevalence in the country and may lead to blindness [31].

According to the World Health Organization, about 80% of eye diseases which may lead to low vision or blindness are preventable or curable. Therefore, an action plan for eye health was suggested in 2014 and aimed to make the ophthalmology services available across the world, to strengthen eye health services in different countries, and to integrate eye health services with other health care services [32]. To date, in order to improve the availability and accessibility of eye health services, telemedicine applications and in particular, teleophthalmology technology has been developed and applied in different countries [15]. Such a service can be considered as a basic ophthalmology service, and regardless of socioeconomic status, it may simply be available to people who are not able to visit a specialist, but need to know his/her opinions. Some of the advantages of this method are saving costs, reducing unnecessary referrals to the specialists, reducing the number of unnecessary travels, and developing effective communication between optometrists and ophthalmologists [33, 34].

While similar studies have focused on the final evaluation of the teleophthalmology systems and paid less attention to the system design process and users’ requirements [35], in this study, optometrists and ophthalmologists were consulted and their opinions were sought to develop and pilot the initial version of the system in several rounds of review to get the final product. It is notable that due to the resource restrictions, the teleophthalmology system was developed using the method of “store and forward”. It was a web-based system and a slit lamp was used to take eye images. Although using a slit lamp allows examining the eye under high magnification, it is expected that new technologies, such as the smart phones and their built-in cameras can also be used in teleophthalmology services in the near future [15].

Despite the advantages of teleophthalmology technology, there are concerns about the quality of images and the accuracy of diagnoses [36]. For example, Kiage *et al*. showed that poor quality images can severely limit the ability of teleglaucoma assessment to diagnose optic nerve damage and glaucoma [37]. However, the results of the current study showed that in most cases, the diagnoses made using the teleophthalmology system was similar to those written in the patients’ records. Moreover, the results of usability testing showed that the users were relatively satisfied with the system’s functions. Even though, there were a number of weaknesses which should be improved in the final product to be able to implement it in other settings.

As the system designed in this study was a web-based system, it was accessible anywhere and anytime *via* the internet. The usability of the system was evaluated by five optometrists and five cornea specialists. They were asked to work with the system, to enter at least two consultation requests, and to complete a standard questionnaire (QUIS) at the end. It was a nine-point likert scale questionnaire and for analyzing data, the scores were divided into three categories: weak (0-3), medium (3.1- 6), and good (6.1- 9).

As Table **1** shows, the mean values for both optometrists and specialists were between 6.1 and 9 in different areas which showed that the users were relatively satisfied with the system. From the specialists’ point of view, the system “interface design” (8.8±0.1) and from the optometrists’ point of view, the system “learnability” (8.7±0.35) had the highest mean values. The lowest mean values were related to “terminology and system information” and the mean values for the specialists and the optometrists were (6.5±0.6) and (7.3±1.7), respectively. To check the accuracy of diagnoses, the diagnoses made by the specialists were compared with the written diagnoses in the patients’ medical records and the results showed that the diagnoses were the same in most cases (Kappa=0.9, 95% CI: 0.71, 1.00). In addition to the above results, some participants believed that the benefits of the system need to be demonstrated before implementing the system in a wider setting. Although the system was easy to use and easy to learn, the users were interested to have more interaction with the system and to receive more messages regarding their interactions. The users were also interested in having access to more system features, such as having access to the medical websites and searching patients, optometrists and ophthalmologists by using their names, ID number or the date of consultation.

## 5 LIMITATIONS

Although it was the first time that a teleophthalmology system was developed in the country, the current study had some limitations. This paper aimed to explain an initial setting up process, which might be useful for other researchers to be able to produce a similar system. Although the initial evaluation of the system showed some weaknesses which can be eliminated in the future versions of the system, organizational and financial supports are needed to develop the final product and to evaluate such a system in a real environment. Therefore, conducting further research by using the final product is recommended.

Another limitation of this study was related to the limited number of the participants. As the researchers aimed to develop a new system, not to generalize the results to a bigger population, it seems that such a small group of participants does not influence the system design process. In fact, the main aim of recruitment was to involve a number of potential users to be able to design a useful system for them.

Due to the time and resources constraints, the researchers focused on only one disease, *i.e*. cataract. However, the application could be tested and expanded for other eye diseases. The system could also be improved by adding more information about post-surgical status of patients. In terms of the usability testing, the final product needs to be used by more users (optometrists and ophthalmologists) to be able to evaluate it in a bigger sample size and show how usable it is from users’ perspectives. Furthermore, the clinical effectiveness of this system was not evaluated in this study due to the resource restrictions. It seems that conducting post-implementation studies with more patients can help to evaluate the impact of the system (*e.g*., reducing unnecessary travels and improving quality of care).

## CONCLUSION

According to the results, it can be concluded that the teleophthalmology technology can be used easily by optometrists and ophthalmologists to improve eye health care services. The use of this technology, especially in the rural or underserved areas can help to prevent or treat many eye diseases in a timely manner. This technology can also be used for screening those who are at the risk of eye diseases, such as cataract and can improve the eye care services in the society.

## Figures and Tables

**Fig. (1):**
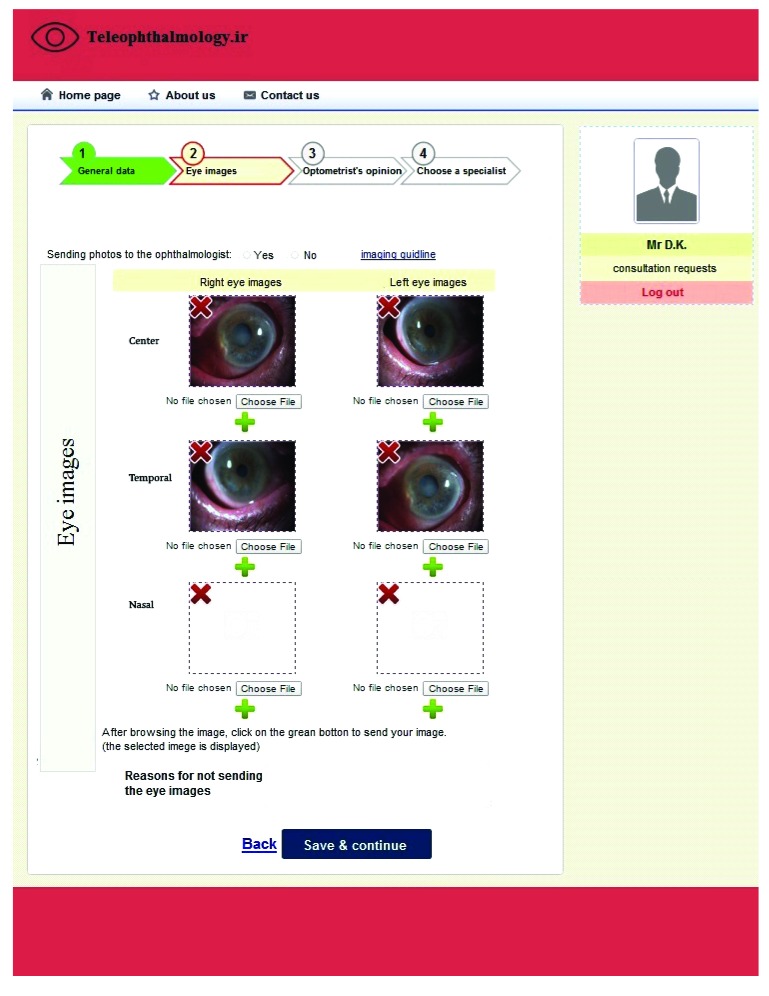
Teleophthalmology consultation request.

**Table 1:** Usability testing of, and users’ satisfaction with, the teleophthalmology system.

Mean ±SD Assessment Areas	Mean ±SD (Ophtalmologists)	Mean ±SD (Optometrists)
Overall reaction to the software	8.2±0.4	7.7 ±1.1
Screen design and layout	8.8±0.1	8.3 ±0.9
Terminology and system information	6.5±0.6	7.3 ±1.7
Learning	8.7±0.3	8.7±0.3
System capabilities	7.3±1.0	7.6 ±1.2
